# Modeling the antidepressant treatment response to transcranial magnetic stimulation using an exponential decay function

**DOI:** 10.1038/s41598-023-33599-w

**Published:** 2023-05-02

**Authors:** Yosef A. Berlow, Amin Zandvakili, McKenna C. Brennan, Leanne M. Williams, Lawrence H. Price, Noah S. Philip

**Affiliations:** 1 Department of Psychiatry and Human Behavior, Alpert Medical School of Brown University, Providence, RI USA; 2 VA RR&D Center for Neurorestoration and Neurotechnology, Providence VA Medical Center, 830 Chalkstone Ave, Providence, RI 02908 USA; 3 Department of Psychiatry and Behavioral Sciences, Stanford University, Stanford, CA USA; 4 Sierra-Pacific Mental Illness Research, Education, and Clinical Center (MIRECC), Veterans Affairs Palo Alto Health Care System, Palo Alto, CA USA; 5 Butler Hospital, Alpert Medical School of Brown University, Providence, RI USA

**Keywords:** Statistical methods, Translational research, Depression

## Abstract

Recovery from depression often demonstrates a nonlinear pattern of treatment response, where the largest reduction in symptoms is observed early followed by smaller improvements. This study investigated whether this exponential pattern could model the antidepressant response to repetitive transcranial magnetic stimulation (TMS). Symptom ratings from 97 patients treated with TMS for depression were collected at baseline and after every five sessions. A nonlinear mixed-effects model was constructed using an exponential decay function. This model was also applied to group-level data from several published clinical trials of TMS for treatment-resistant depression. These nonlinear models were compared to corresponding linear models. In our clinical sample, response to TMS was well modeled with the exponential decay function, yielding significant estimates for all parameters and demonstrating superior fit compared to a linear model. Similarly, when applied to multiple studies comparing TMS modalities as well as to previously identified treatment response trajectories, the exponential decay models yielded consistently better fits compared to linear models. These results demonstrate that the antidepressant response to TMS follows a nonlinear pattern of improvement that is well modeled with an exponential decay function. This modeling offers a simple and useful framework to inform clinical decisions and future studies.

## Introduction

Major depressive disorder is the leading cause of disability worldwide^1^ and represents a common risk factor for poor clinical outcomes. Unfortunately, many individuals with major depression do not respond to conventional pharmacological interventions^2^. Repetitive transcranial magnetic stimulation (TMS) offers a noninvasive nonpharmacological treatment option that is effective for treatment-resistant depression^3^. This therapeutic approach uses repetitive pulsed magnetic fields to induce depolarization in brain regions that modulate neural networks and alter pathological patterns of brain activity seen in depression^4^. TMS lacks the systematic side effects associated with pharmacological antidepressant treatments and is generally well tolerated and safe. Furthermore, there is emerging literature supporting the use of TMS for a wide range of neuropsychiatric disorders for which pharmacotherapy offers limited efficacy (reviewed in Refs.^5,6^).

Despite this therapeutic promise, TMS remains an underutilized psychiatric treatment. Challenges limiting more widespread adoption of TMS include the time commitment for patients and clinicians and variable responses to treatment. A standard course of TMS requires daily in-clinic treatments for thirty sessions, often followed by a taper series^3^. Standard treatment delivery limits the number of patients that can be treated on a single device to 60–80 per year, making TMS a limited resource that can only be administered to a small percentage of individuals with treatment-resistant depression. While recent studies have suggested that faster responses might be obtained with additional daily sessions, this literature remains mixed, and the time commitments are still substantial (e.g.^7–9^). Furthermore, response to TMS is variable, as some patients respond rapidly and robustly, whereas others demonstrate slower responses or no response at all^10,11^.

Several studies have investigated the potential of utilizing neuroimaging techniques to develop biomarkers of TMS response that could identify patients more likely to respond^12–14^. However, these assessments remain exploratory research tools that are challenging to implement and have not yet demonstrated clinical utility^15^. There have also been efforts to use early symptomatic improvement to predict a later response to TMS^16,17^, but findings have been mixed when examined in different settings^18^. To this end, a generalized model of TMS treatment response could inform the testing and development of measurement-based care strategies to optimize outcomes and identify patients more likely to respond to additional TMS treatments, thus increasing the percentage of successful courses of treatment.

Pharmacological antidepressant treatments have been shown to exhibit a nonlinear pattern of treatment response, with relatively large improvements in symptoms in the first weeks followed by smaller but continued improvements approaching a plateau^19–21^. This decrease in depressive symptoms has been modeled using an exponential decay function across multiple medications^22–24^. The exponential decay model suggests a defined relationship between early clinical response and eventual treatment outcomes. However, it is unclear if this model of treatment response can be extrapolated to other modalities, such as TMS for treatment-resistant depression.

The objectives of this study were to investigate whether an exponential decay function could model the treatment response to TMS using a clinical cohort sample and a broad range of recent TMS studies for treatment-resistant depression from around the world, and to test whether this model could predict clinical outcomes based on early clinical response. This line of inquiry aims to develop a framework for understanding the pattern of recovery from depression with TMS to inform clinical decisions and future protocols.

## Methods

### Overview

We report on data from our retrospective clinical sample of 97 patients undergoing TMS for treatment resistant depression, as well as novel modeling analyses of data from published clinical trials of TMS protocols. Primary outcome measures were longitudinal ratings of depressive symptoms acquired during TMS protocols for treatment resistant depression. The primary analyses utilize nonlinear mixed-effects models (NLME) to apply the exponential decay function to individual-level and group-level data of TMS treatment response and compare these nonlinear models against linear models. Additionally, we assess the predictive utility of rearranging the exponential decay function to predict treatment outcomes based on early response.

### Participants and data acquisition

De-identified symptom rating data were collected from a naturalistic sample of 97 patients treated with clinical TMS at the VA Providence Healthcare System (Providence, RI, USA). Demographics were representative of the VA population with an average age of 53.76 years (sd = 13.20) and majority (86%) male. Stimulation protocols followed clinical practice, with most patients receiving 10 Hz TMS delivered to the left dorsolateral prefrontal cortex. TMS was administered every weekday for up to 30 sessions, followed by six taper sessions. Symptom ratings were assessed using the Patient Health Questionnaire 9 (PHQ-9)^25^ at baseline and after every five TMS treatment sessions. The PHQ-9 is a standard clinical rating scale for depression that provides adequate information about symptom severity and minimizes rater burden, with established psychometrics that facilitate its comparison with other depression rating scales. Scores range from 0 to 27, with higher scores indicative of more severe depression.

Group-level symptom rating data were also collected from published clinical trials investigating various TMS protocols. These studies included (1) a randomized noninferiority study comparing Theta Burst Stimulation (TBS) (n = 193) to standard 10 Hz stimulation (n = 192)^26^, (2) a randomized controlled trial comparing standard and high-dose protocols of left-sided 10 Hz stimulation (standard n = 59, high-dose n = 91) and right-sided 1 Hz stimulation (standard n = 57, high-dose n = 93)^27^, (3) a randomized controlled trial comparing supra- (120% motor threshold (MT)) and sub-threshold (80% MT) accelerated bilateral TBS (suprathreshold n = 87, subthreshold n = 93) to standard left-sided 10 Hz stimulation (n = 72)^9^, (4) and an unblinded trial of accelerated, high-dose TBS using functional MRI guided targeting (n = 20)^7^. These studies contribute heterogenous data incorporating a variety of TMS paradigms, supporting the generalizability and relevance of our analysis. Additional group-level symptom rating data were collected from a secondary analysis of the THREE-D Study^26^ in which unique group-level treatment response trajectories were identified in subjects receiving either left-sided 10 Hz TMS or left-sided TBS (n = 388)^10^. Primary outcome measures in these studies were the Hamilton Depression Rating Scale (HAM-D)^28^ (17 item and 6 item), and the Quick Inventory of Depressive Symptoms Clinician-Rated (QIDS-C)^29^. Longitudinal group-level mean symptom rating data were obtained from the included tables^9,27^ or extracted from figures using http://www.graphreader.com/^7,10,26^.

### Statistical analyses

**Figure 1:**
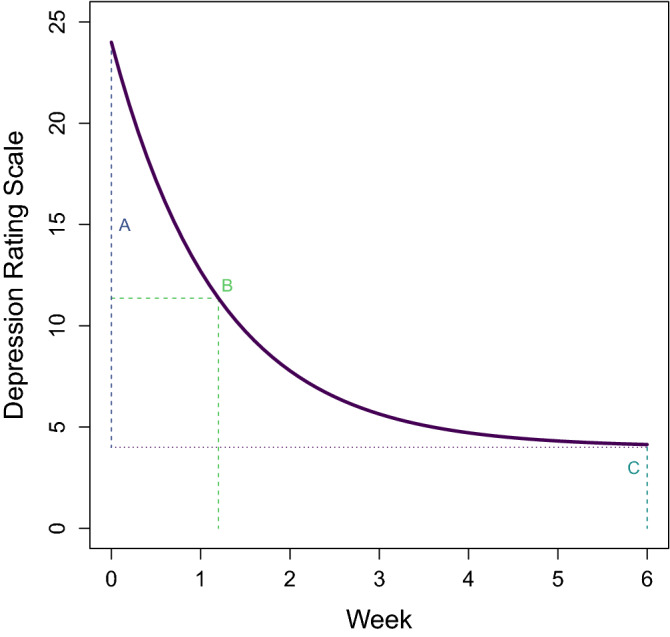
Exponential decay model of the treatment response to transcranial magnetic stimulation (TMS). Change in depression ratings during TMS are expressed as a function of time (*t*), with *A* representing the total magnitude of symptom improvement, *B* representing the time constant of treatment response, and *C* representing symptom ratings at the end of treatment.

The TMS treatment response was modeled using the exponential decay function in Eq. (1):

1 $$\begin{array}{c}D\left(t\right)=A\times {e}^{\left(\frac{-t}{B}\right)}+C ,\end{array}$$

in which symptom ratings (*D*) at time *t* are described using the magnitude of total response (*A*), decaying at a time constant (*B*), and approaching a minimum value after treatment completion (*C)* as shown in Fig. 1. In this model, parameters *A* and *C* are in the units of the symptom rating scale (i.e., score) and parameter *B* is time in weeks.

NLME models were constructed for our naturalistic sample receiving clinical TMS using Eq. (1). Model parameters were treated as random effects at the patient level and as fixed effects at the group level. A model treating all parameters (*A*, *B*, and *C*) as random effects was compared to the simpler model fitting only parameters *A* and *C* as random effects. The simpler model provided an equivalent fit as assessed by the likelihood ratio test (LRT) and lower Akaike information criterion (AIC) and Bayesian information criterion (BIC) values, and this simpler model was chosen. This NLME model was compared to a corresponding linear mixed-effect (LME) model in which slope and intercept were treated as random effects at the patient level using the AIC, BIC, and LRT. The predictive utility of this model was assessed using k-fold (k = 5 and k = 10) and leave-one-out cross-validation (LOOCV), estimating the time constant (*B*) on a subset of the sample and using these estimates to calculate predicted treatment outcomes (*C*) for the left-out sample based on symptom rating scores at baseline and after five or ten TMS sessions.

Similar NLME models were constructed using the exponential decay function in Eq. (1) for three published group-level studies comparing various TMS protocols^9,26,27^ with parameters A and C treated as random effects at the treatment group level. As above, these simpler models provided equivalent fits when compared to models using all parameters as random effects. These NLME models were subsequently compared to corresponding LME models in which the slope and intercept were treated as random effects at the group level using AIC, BIC, and LRT.

The exponential decay model was also fit at the group level using nonlinear least squares for each treatment group in the above studies^9,26,27^ as well as the unblinded trial of accelerated, high-dose TBS^7^. Corresponding linear models were compared to nonlinear exponential decay models using AIC and BIC.

Finally, a NLME model was constructed using the exponential decay function for the unique group-level symptom trajectories previously identified in 388 subjects receiving either left-sided 10 Hz TMS or left-sided TBS^10^. Here, a model incorporating all parameters (*A*, *B*, *C*) as random effects yielded significant LRT and lower AIC and BIC values compared with a simpler NLME using only parameters *A* and *C* as random effects. This NLME model was then compared to a corresponding LME model in which slope and intercept were considered random effects at the group level using AIC, BIC, and LRT.

All mixed effect models were fit using maximum likelihood as implemented in the Nonlinear Mixed-Effects Models library in R^30^.

### Ethics declaration

This study was performed in accordance with the Declaration of Helsinki. The procedures for gathering and analyzing de-identified clinical data were approved as quality improvement by the VA Palo Alto/Stanford Institutional Review Board^31^. Additional group-level analyses were based on publicly available data from previously published studies^7,9,10,26,27^.

## Results

### Modeling treatment response to TMS in a clinical sample

**Figure 2:**
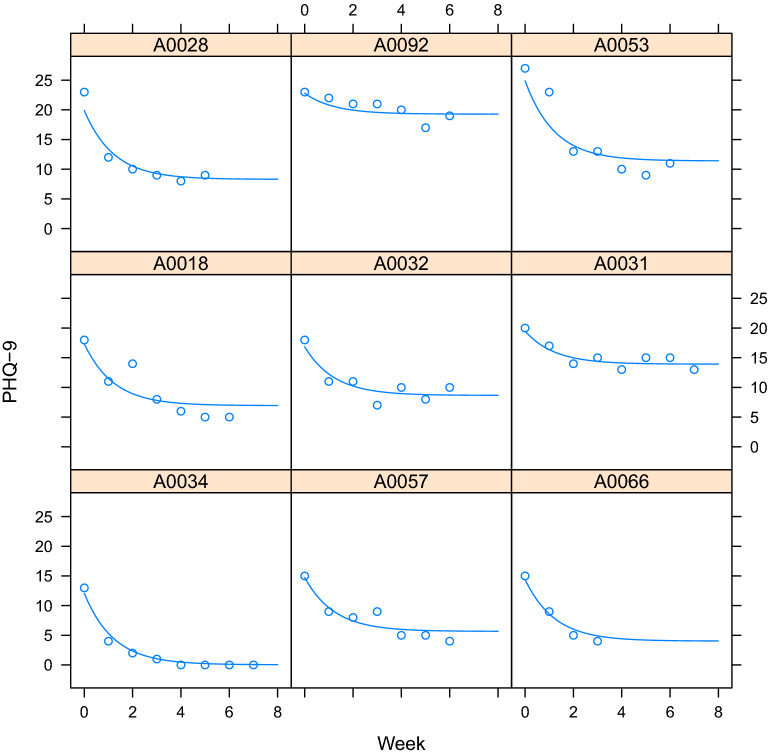
Individual estimates of TMS treatment response from the nonlinear mixed-effects model. These panels illustrate representative longitudinal depression ratings from the Patient Health Questionnaire 9 (PHQ-9) during TMS treatment (open circles) from nine individuals as well as the nonlinear mixed-effects model fit (line).

The NLME model demonstrated that the TMS treatment response was well modeled with the exponential decay function, yielding significant estimates for model parameters *A*, *B*, and *C* (all p < 0.0001). Each individual contributed 2 to 9 (median = 6) longitudinal measurements of depressive symptoms for a total of 562 observations. The exponential model captured a wide range of individual treatment response trajectories, as illustrated in Fig. 2. The magnitude of total response (*A*) was estimated to be a clinically meaningful 5.8-point drop in PHQ-9 scores with a time constant (*B*) of 1.2 weeks or 6 TMS treatments. When compared to a corresponding LME, the exponential decay model displayed lower AIC and BIC values and a significant likelihood ratio (LRT = 63.2, p < 0.0001), suggesting that the NLME model based on the exponential decay function is a better fit.

### Predicting treatment outcomes to TMS based on early response

In subsets containing complete longitudinal data from this naturalistic sample, k-fold and leave-one-out cross-validation approaches yielded consistent estimates of the time constant *B* (fivefold: mean 1.218 weeks, sd 0.079; tenfold: mean 1.217 weeks, sd 0.080; LOOCV: mean 1.213 weeks, sd 0.023). By rearranging Eq. (1) as shown in Eq. (2), we calculated predicted values of *C* for the left-out samples using the time constant estimate (*B*) and symptom scores at baseline and after one week (n = 90) or two weeks (n = 82) of treatment. Predicted C values were constrained to 0 to 27, inclusive, corresponding with the range of possible PHQ-9 scores.

2 $$\begin{array}{c}\frac{D\left(t\right)-D\left(0\right){\times e}^{\left(\frac{-t}{B}\right)}}{1-{e}^{\left(\frac{-t}{B}\right)}}=C.\end{array}$$

Predicted C values from each cross-validation approach were highly correlated (r > 0.999) and yielded significant correlations with PHQ-9 scores at the end of treatment, accounting for 38% to 58% of the variance in final scores, using PHQ-9 scores at baseline and after one week (adjusted R^2^ = 0.379 to 0.383, p < 0.0001) or two weeks (adjusted R^2^ = 0.576 to 0.580, p < 0.0001) of daily TMS sessions.

Predicted categorical treatment response, defined by a 50% reduction in PHQ-9 scores at the end of treatment, was assigned using the C values in Eq. (2) (i.e. C ≤ 0.50 × D(0)). These assignments did not differ across cross-validation methods. Using week 1 predictions, treatment response was assigned with 78% accuracy, 68% sensitivity, 82% specificity, 85% negative predictive value (NPV), and 63% positive predictive value (PPV). Assigning categorical treatment response using week 2 estimates, yielded 80% accuracy, 73% sensitivity, 84% specificity, 87% NPV and 68% PPV.

### Modeling group-level treatment response patterns to TMS

The NLME model was extended to group-level data from three published studies comparing multiple TMS modalities for treatment-resistant depression^9,26,27^, including left-sided high-frequency (10 Hz) repetitive TMS, standard and accelerated TBS, and right-sided, low-frequency (1 Hz) stimulation. These NLME models using the exponential decay function yielded significant estimates for all model parameters (all p < 0.005).

In a randomized noninferiority study comparing TBS to 10 Hz stimulation, the “THREE-D” study^26^, the NLME model estimated the magnitude of total response (*A*) to be a drop of 12.2 points on the HAM-D with a time constant of 2.3 weeks. When compared to the corresponding LME model, the NLME model yielded a significant likelihood ratio (LRT = 18.1, p < 0.0001) and lower AIC (49 vs 65) and BIC values (53 vs 69), demonstrating the NLME model is a better fit.

Similarly, when this exponential decay model was applied to a randomized controlled trial comparing left-sided 10 Hz stimulation and right-sided 1 Hz stimulation^27^, estimates of the treatment response (*A*) demonstrated an average 13.6 point drop on the HAM-D with a time constant B of 1.2 weeks. This NLME model demonstrated a significant likelihood ratio (LRT = 22.7, p < 0·0001) and lower AIC (81 vs. 102) and BIC (88 vs. 108) values compared to the corresponding LME model, indicating the nonlinear model is a better fit.

Finally, when applied to a randomized controlled trial comparing accelerated bilateral TBS above (120%) and below (80%) MT to standard left-sided 10 Hz stimulation^9^, the NLME model yielded estimates of a total 6.0 point drop on the QIDS-C^29^ with a time constant of 0.7 weeks. When compared to the corresponding LME model, the NLME model yielded a significant likelihood ratio (LRT = 44.1, p < 0.0001) and lower AIC (30 vs 72) and BIC (35 vs 76) values, suggesting the exponential decay model was a superior fit.

**Figure 3:**
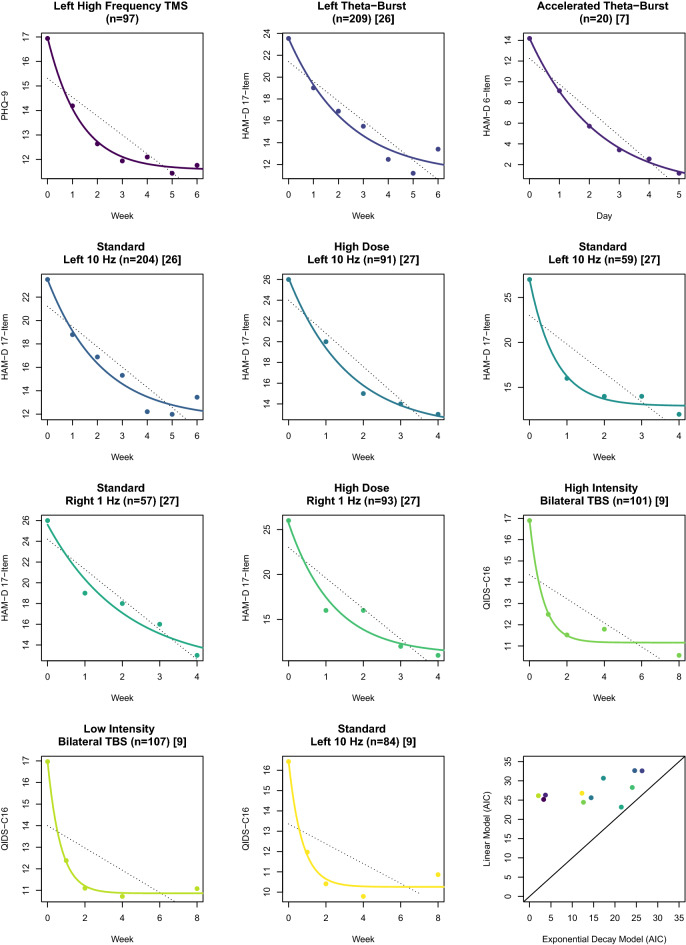
Modeling group-level symptom response to transcranial magnetic stimulation (TMS): the exponential decay model was applied to group-level depression ratings from our clinical sample (n = 97) as well as group-level data four published clinical trials^7,9,26,27^ utilizing a variety of TMS protocols including left-sided repetitive 10 Hz TMS, standard and accelerated theta-burst stimulation, and right-sided, low-frequency (1 Hz) TMS. In each case, the exponential decay models (solid line) yielded better fits when compared to corresponding linear models (dotted line) as illustrated by lower Akaike Information Criterion (AIC) values (lower right).

The exponential decay model was also fit at the individual group level using nonlinear least squares for each group in the above studies^9,26,27^, an unblinded accelerated, high dose TBS study^7^ and our naturalistic clinical sample. Although these individual group fits did not always yield significant estimates for all model parameters based on limited degrees of freedom, when the AIC and BIC values from these nonlinear fits were compared to corresponding linear models of the same group-level data, exponential decay models consistently yielded lower AIC and BIC values for all TMS samples studied (Fig. 3).

### Modeling unique group-level response trajectories to TMS

**Figure 4:**
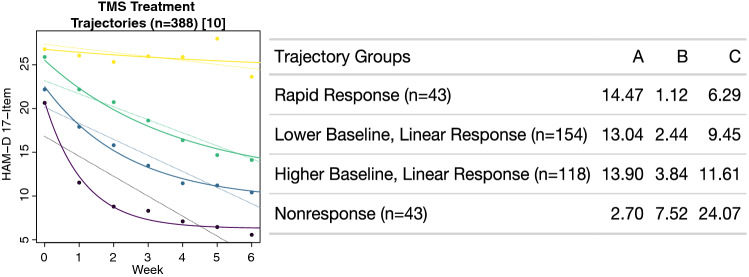
Modeling unique treatment response trajectories to transcranial magnetic stimulation (TMS): A nonlinear mixed-effects model (solid lines) was constructed to model the four treatment trajectories identified by Kaster et al. (N = 388)^10^. Each of these response trajectories could be described using the three parameters of the exponential decay function (Eq. 1). This nonlinear mixed-effects model demonstrated a superior fit when compared to a corresponding linear mixed-effects model (dotted lines).

When the exponential decay model was applied to the unique group-level symptom trajectories identified in the secondary analysis of the THREE-D study^10^, the NLME model was able to describe each group trajectory in terms of parameters *A, B*, and *C*, (Eq. 1, Fig. 4). The three groups of responders demonstrated similar estimates of the magnitude of treatment response (parameter *A*), with reductions of 13.0, 13.9 and 14.5 points on the HAM-D for “Lower baseline symptoms, linear response”, “Higher baseline symptoms, linear response”, and “Rapid response” groups, respectively. However, these groups differed more markedly in their group estimates of the time constant *B*, with “Rapid response” at 1.1 weeks, followed by “Lower baseline symptoms, linear response” at 2.4 weeks, and “Higher baseline symptoms, linear response” at 3.8 weeks. These differences correspond to the speed at which these groups approach their minimum depressive symptom rating scores during TMS treatment. In contrast, the “Nonresponse” group was associated with a minimal estimate of total treatment response (parameter A) of a 2.7-point reduction on the HAM-D and a long estimate for the time constant B of 7.5 weeks. This NLME model was associated with a significant likelihood ratio (LRT = 30.7, p < 0.0001) and lower AIC (121 vs. 143) and BIC (134 vs. 151) values compared to a corresponding LME model, indicating that these trajectories were better modeled with the nonlinear exponential decay function.

## Discussion

These results provide substantial evidence that the antidepressant response to TMS demonstrates a nonlinear pattern of improvement that follows an exponential decay function. This pattern of early large reductions in depressive symptom ratings followed by smaller improvements is similar to the trajectory seen with pharmacological depression treatments^19–23^. This pattern is further replicated at the group level in multiple clinical TMS trials utilizing various stimulation protocols and clinical rating scales representing over 1000 individuals^7,9,26,27^. This study also demonstrated that this model can provide a parsimonious method to describe a variety of treatment response trajectories to TMS, including those previously identified using group-based trajectory modeling^10^. Finally, this study provides evidence that the exponential decay model can be applied to define and predict individual treatment response outcomes based on early clinical response.

An advantage of this nonlinear modeling approach is that it provides fitted parameters that have real-world meaning. In this model, *A* is the total magnitude of response as measured by a point reduction in clinical rating scales, *B* is the time constant of the response representing the speed of recovery as a function of time, and *C* is the minimum expected symptom rating at the end of treatments. This simple quantification of the magnitude, speed, and outcome of TMS treatment response provides a comprehensive estimate of the treatment course that can be readily translated into common metrics of clinical outcomes for depression treatment; categorical treatment response (50% reduction in symptoms) would occur when *A* is greater than or equal to *C*, and remission would occur when *C* is below a rating scale threshold (e.g., PHQ-9 < 5). Additionally, this model incorporates the relationship between the speed of improvement and length of TMS treatment, providing a formula that relates early treatment response to eventual treatment outcomes. At time *B*, one would expect the magnitude of treatment response to reflect ~ 63% (1–1/e) of the total response. At time 3x*B*, this would reflect 95% (1–3/e) of the total expected response. While most of the groups included in this study only incorporated time points during TMS treatment, the study by Chen et al.^9^ also included longitudinal measurements weeks after treatment, implying estimates of *C* may extend to post-treatment outcomes.

Using our clinical TMS sample, we demonstrated that one could use a population average value for the time constant *B* to predict individual treatment outcomes based on the response at weeks one or two. This work is consistent with previous studies demonstrating that early TMS treatment response can predict later treatment outcomes^16,17^. However, it is unlikely a single time constant will describe all individuals’ treatment response patterns adequately. Additional studies using well-sampled longitudinal measurements integrating this variability in speed of recovery are needed to further elucidate the utility of this model at the individual level. At the group-level, we found evidence that the time constant of recovery may differ based on the population, treatment protocol, and the depression rating scale used. Additionally, applying this model to the group trajectories identified by Kaster et al.^10^ suggests that variability in the speed of recovery reflected by this time constant is a key difference defining the unique group and, by extrapolation, individual treatment response trajectories. For an individual undergoing TMS, one could estimate this time constant using nonlinear least squares to fit the exponential decay model to a target outcome (C) to determine whether a patient could benefit from additional TMS treatments given slower early response in concordance with current clinical recommendations^32^.

The limitations of this work are its use of available datasets and retrospective analysis; future research will be needed to test whether these findings can be used prospectively to determine optimal treatment courses. Furthermore, while TMS, given its resource-intensive nature, is an appropriate context for developing this model, novel pharmacotherapies and psychotherapies for depression could also benefit from application of the model. Whether and how these models differ from placebo response will be an important area of future inquiry. These limitations aside, these data clearly demonstrate the nonlinear trajectory of symptom improvement with TMS for treatment resistant depression across many different protocols and illustrate methods to predict treatment outcomes based on early response. This modeling offers a simple and useful framework to describe the antidepressant response to TMS that could inform clinical decisions and future studies.

## Data Availability

Data from the clinical sample and analysis code are available from the corresponding author on reasonable request. Group-level data are available in the main text and in the text of previously published studies^7,9,10,26,27^.
